# Challenges in the diagnosis and treatment of pure non-gestational uterine choriocarcinoma in a child: a case report

**DOI:** 10.1186/s13256-024-04664-3

**Published:** 2024-07-15

**Authors:** Faisol Darmawan, Aditya Rifqi Fauzi, Rogatianus Bagus Pratignyo, Pieri Kumaladewi, Andrini Esha Rahmadanty, Andreas Niko Santhony, Hanggoro Tri Rinonce, Gunadi

**Affiliations:** 1 Pediatric Surgery Division, Department of Surgery, Faculty of Medicine, University of Lampung/RSUD Abdul Moeloek, Bandar Lampung, 35141 Indonesia; 2 Pediatric Surgery Division, Department of Surgery, Faculty of Medicine, Public Health and Nursing, Universitas Gadjah Mada/Dr. Sardjito Hospital, Jl. Kesehatan No. 1, Yogyakarta, 55281 Indonesia; 3 Department of Child Health, Faculty of Medicine, University of Lampung/RSUD Abdul Moeloek, Bandar Lampung, 35141 Indonesia; 4 Department of Anatomical Pathology, Imanuel Way Halim Hospital, Bandar Lampung, Lampung 35141 Indonesia; 5 Department of Anatomical Pathology, Faculty of Medicine, Public Health and Nursing, Universitas Gadjah Mada/Dr. Sardjito Hospital, Yogyakarta, 55281 Indonesia

**Keywords:** Case report, Challenges diagnosis and treatment, Chemotherapy, Non-gestational uterine choriocarcinoma prognosis, Pediatric patient, Surgery

## Abstract

**Background:**

Diagnosing non-gestational uterine choriocarcinoma in children is challenging because of its rarity and nonspecific imaging findings. Herein, we report a case of non-gestational uterine choriocarcinoma in a child, which was unexpectedly found during exploratory laparotomy and confirmed by histopathological findings. However, the tumor did not respond to chemotherapy.

**Case presentation:**

A 4-year-old Indonesian female patient was brought into the emergency unit with chief complaint of vaginal bleeding. She had suffered from vaginal spotting 4 months before being admitted to the hospital. Physical examination revealed a distended abdomen in the left lumbar region and a palpable fixed mass with a smooth surface. Abdominal computed tomography scans revealed a large mass (10 × 6 × 12 cm) with fluid density and calcification. Thus, we suspected left ovarian teratoma. The patient’s luteinizing hormone, follicle-stimulating hormone, and lactate dehydrogenase levels were 25.2 mIU/ml, 0.1 mIU/ml, and 406 U/l, respectively. According to the clinical and radiological findings, we decided to perform an exploratory laparotomy and found a tumor originating from the uterus, not the ovarium. We did not observe liver nodules and any enlargement of abdominal lymph nodes. Subsequently, we performed hysterectomy. The histopathological findings supported the diagnosis of choriocarcinoma. The patient was discharged uneventfully on postoperative day 5. Thereafter, the patient underwent nine cycles of chemotherapy, including carboplatin (600 mg/m^2^ IV), etoposide (120 mg/m^2^ IV), and bleomycin (15 mg/m^2^ IV). However, on the basis of the clinical findings of a palpable mass and partial intestinal obstruction, the tumor relapsed soon after the ninth cycle of chemotherapy. Currently, the patient is undergoing chemotherapy again.

**Conclusions:**

Although pure non-gestational uterine choriocarcinoma is rare, it should be considered as one of the differential diagnoses for intraabdominal tumors in a child, so as to better guide and counsel families regarding the surgical plan and prognosis, respectively. In the present case, the patient’s response to chemotherapy was poor, implying that the treatment of non-gestational choriocarcinoma is still challenging, particularly in the pediatric population.

## Background

Gestational choriocarcinoma (GC) is a malignant tumor that frequently develops in the uterus during pregnancy. Its clinical diagnosis is relatively easy because of the significant association between pregnancy and high levels of β-human chorionic gonadotropin (β-hCG) [1]. However, diagnosing non-gestational uterine choriocarcinoma in children is challenging because of its rarity and nonspecific imaging findings [2].

GC and non-gestational GC (NGC) have different prognoses, with the latter having poorer prognosis [1]. Herein, we report a case of non-gestational uterine choriocarcinoma in a child, which was unexpectedly found during exploratory laparotomy and confirmed by histopathological findings. However, the tumor did not respond to chemotherapy.

## Case presentation

**Fig. 1:**
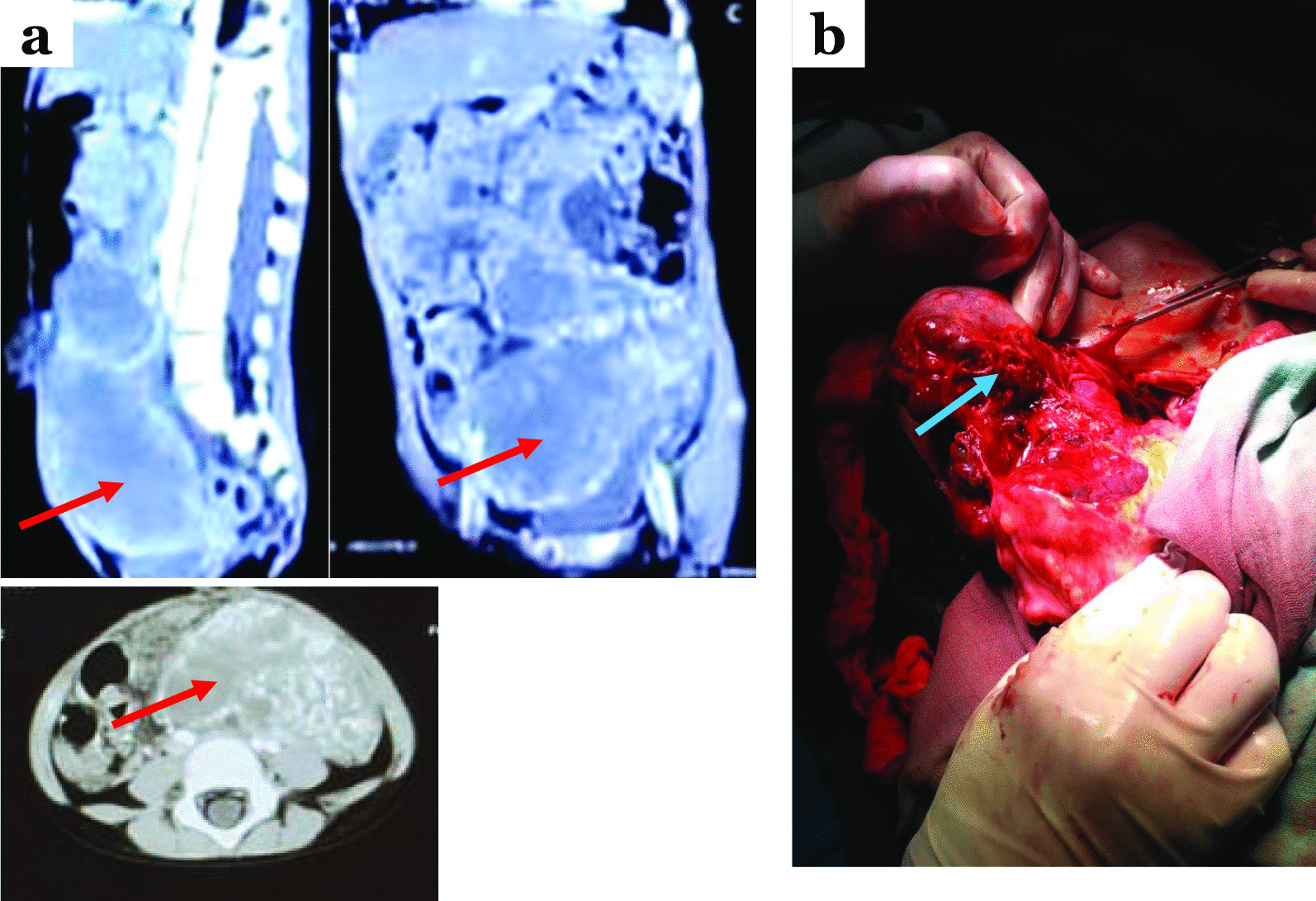
**a** Abdominal computed tomography scan showing a large mass (10 × 6 × 12 cm) with fluid density and calcification (red arrow), leading to the suspicion of left ovarian teratoma. **b** Intraoperative finding revealing a tumor originating from the uterus (blue arrow), not the ovarium

**Fig. 2:**
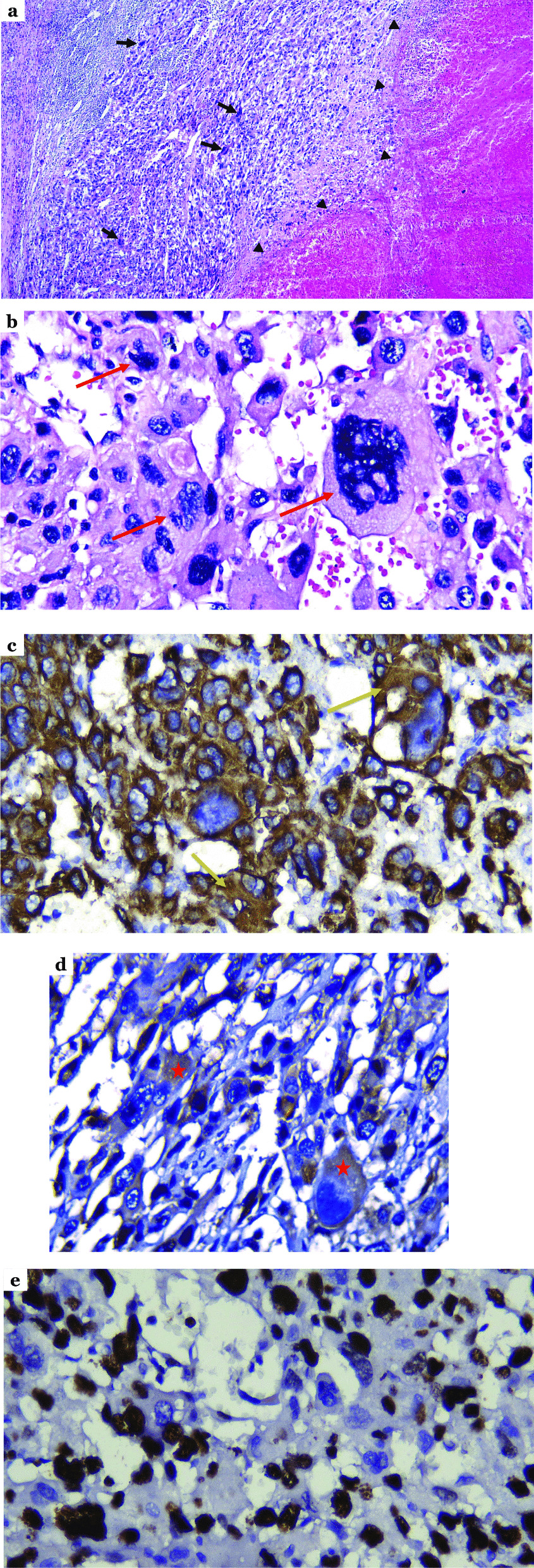
Microscopic examination showing that the tumor comprised diffusely infiltrative intermediate trophoblasts, cytotrophoblasts, and syncytiotrophoblasts (black arrow) and the presence of significant hemorrhage and necrosis (black arrowhead) (**A** hematoxylin–eosin, 40×). The tumor cells show striking cytologic atypia (red arrow) (**B** hematoxylin–eosin, 400×). Moreover, they diffusely express cytokeratin AE1/AE3 (yellow arrow) [**C**] and focally express β-human chorionic gonadotropin (red star) [**D**] in their cytoplasm on immunohistochemistry (400×). Ki-67 immunostaining [**E**] shows a high proliferative index (74%)

A 4-year-old Indonesian female patient was brought into the emergency unit with chief complaint of vaginal bleeding. She had suffered from vaginal spotting 4 months before being admitted to the hospital and lost 4 kg in weight. No family history of cancer was noted. Physical examination revealed a distended abdomen in the left lumbar region and a palpable fixed mass with a smooth surface. Abdominal computed tomography (CT) scans revealed a large mass (10 × 6 × 12 cm) with fluid density and calcification. Thus, we suspected left ovarian teratoma (Fig. 1a). The patient’s luteinizing hormone, follicle-stimulating hormone, and lactate dehydrogenase levels were 25.2 mIU/ml, 0.1 mIU/ml, and 406 U/l, respectively. Unfortunately, we could not obtain β-hCG data preoperatively. According to the clinical and radiological findings, we decided to perform an exploratory laparotomy and found a tumor originating from the uterus, not the ovarium (Fig. 1b). We did not observe liver nodules and any enlargement of abdominal lymph nodes. Subsequently, we performed hysterectomy. Histopathological examination showed that the tumor comprised diffusely infiltrative atypical intermediate trophoblasts, cytotrophoblasts, and syncytiotrophoblasts, with numerous mitotic figures. There were significant hemorrhage and necrosis, and chorionic villi were absent. Immunohistochemistry examination revealed diffuse cytoplasmic expression of cytokeratin AE1/AE3 and focal cytoplasmic expression of human chorionic gonadotropin, particularly in syncytiotrophoblasts. In addition, the tumor showed a proliferative index of 74% (Fig. 2). The results of CD30 immunostaining were negative, excluding the differential diagnosis of embryonal carcinoma. Thus, the final diagnosis was uterine choriocarcinoma.

The patient was discharged uneventfully on postoperative day 5. Thereafter, the patient underwent nine cycles of chemotherapy, including carboplatin (600 mg/m^2^ IV), etoposide (120 mg/m^2^ intravenous), and bleomycin (15 mg/m^2^ intravenous). However, on the basis of the clinical findings of a palpable mass and partial intestinal obstruction, the tumor relapsed soon after the ninth cycle of chemotherapy. Currently, the patient is undergoing chemotherapy again.

## Discussion

NGC is usually found in the ovaries and rarely in the uterus [1]. NGC has been reported in children, particularly infants [3]. Most cases of infantile choriocarcinoma are metastasis from intraplacental choriocarcinoma to the fetus [3]. Infantile choriocarcinoma usually has an onset of 1 month [2]. Its typical symptoms include anemia, developmental delay, hepatomegaly, hemoptysis, or respiratory failure [3]. However, this was not the case for our patient. In our case, the patient presented with vaginal bleeding as the first symptom at the age of 4 years. To the best of our knowledge, our case is the first report of uterine choriocarcinoma in a child, not an infant or neonate. Currently, 30 cases of infantile choriocarcinoma have been reported [2].

Infantile choriocarcinoma is an exceedingly rare malignant tumor. In a previous study, all 30 cases of infantile choriocarcinoma revealed elevated β-hCG levels [2]. However, the diagnosis of infantile choriocarcinoma is usually incorrect because of its rarity, nonspecific imaging findings, and the absence of known maternal choriocarcinoma [4, 5]. Unfortunately, the β-hCG level in our case was not determined because of limited hospital resources. Nevertheless, immunohistochemical analysis of our case confirmed that the tumor cells expressed β-hCG in their cytoplasm (Fig. 2d).

The pathogenesis of NGC remains unclear. However, the following hypotheses have been proposed: (1) the retained totipotent germ cells undergo anomalous migration at the embryo stage, apoptosis failure occurs, and the cells change into a choriocarcinoma and (2) adult cells undergo dedifferentiation, resulting in different morphological cells, such as trophoblastic and non-trophoblastic cells [6, 7].

Of note, the diagnosis in our case was unexpectedly determined during exploratory laparotomy. The preoperative diagnosis was missed because the computed tomography (CT) scan suggested that the tumor originated from the ovarium, not the uterus (Fig. 1a). These difficulties in establishing preoperative diagnosis might be because uterine choriocarcinoma is rare and disease onset did not occur during infancy. Moreover, infantile choriocarcinoma has a very poor prognosis, and delays in diagnosis result in a high mortality rate. Without appropriate management, the patient might die within 3 weeks after the first symptoms [8]. Moreover, we were unable to perform exploratory laparoscopy or magnetic resonance imaging (MRI) because of unavailability in our institution. MRI has several advantages in evaluating pelvic mass, particularly in children, including incredible soft tissue contrast resolution and absence of ionizing radiation [9]. Furthermore, if a solid uterine mass is determined preoperatively, an ultrasound-guided tru-cut biopsy can be performed [10]. In addition, one differential diagnosis of our case is endometrial cancer (EC), since most patients with EC suffer from abnormal bleeding from the vagina [11]. However, the symptom mostly occurs during the period of post-menopause [11], which was not the case here. They suggest examining the endometrial histopathology and MRI for a precise diagnosis [11].

Our patient did not respond to chemotherapy. NGC has been reported to be resistant to chemotherapy [12, 13]. Moreover, a previous study showed that the survival rate of infantile choriocarcinoma is only 17% [2]. There is no standard treatment for NGC. Thus, NGC treatment is based on the management for GC [1]. Surgery is essential for NGC treatment since the neoplasm is derived from the patient [1].

## Conclusion

Although pure non-gestational uterine choriocarcinoma is very rare, it should be considered as one of the differential diagnoses for intraabdominal tumors in a child, so as to better guide and counsel families regarding the surgical plan and prognosis, respectively. Moreover, in the present case, the patient’s response to chemotherapy was poor, implying that the treatment of NGC is still challenging, particularly in the pediatric population.

## Data Availability

All data generated or analyzed during this study are included in the submission.

## Abbreviations

β-hCG: β-Human chorionic gonadotropin
CT: Computed tomography
GC: Gestational choriocarcinoma
MRI: Magnetic resonance imaging
NGC: Non-gestational choriocarcinoma
